# Canine Lafora Disease: An Unstable Repeat Expansion Disorder

**DOI:** 10.3390/life11070689

**Published:** 2021-07-14

**Authors:** Thilo von Klopmann, Saija Ahonen, Irene Espadas-Santiuste, Kaspar Matiasek, Daniel Sanchez-Masian, Stefan Rupp, Helene Vandenberghe, Jeremy Rose, Travis Wang, Peixiang Wang, Berge Arakel Minassian, Clare Rusbridge

**Affiliations:** 1 Department of Neurology, Small Animal Clinic Hofheim, 65719 Hofheim, Germany; t.vonklopmann@tierklinik-hofheim.de (T.v.K.); s.rupp@tierklinik-hofheim.de (S.R.); 2 Program in Genetics and Genome Biology, The Hospital for Sick Children, Toronto, ON M5G 0A4, Canada; saija.ahonen@gmail.com (S.A.); travisjwang@gmail.com (T.W.); peixiang.wang@sickkids.ca (P.W.); 3 Institute of Veterinary Science, University of Liverpool, Leahurst Campus, Chester High Road, Neston CH64 7TE, UK; Irene.Espadas@scarsdalevets.com (I.E.-S.); dsanchezmasian@gmail.com (D.S.-M.); 4 Section of Clinical and Comparative Neuropathology, Ludwig-Maximilians-Universität München, 80539 München, Germany; kaspar.matiasek@neuropathologie.de; 5 Highcroft Veterinary Referrals, Whitchurch, Bristol BS14 9BE, UK; Helene.Vandenberghe@highcroftvet.co.uk; 6 Lumbry Park Veterinary Specialists, Alton GU34 3HL, UK; Jeremy.Rose@cvsvets.com; 7 Division of Neurology, Department of Pediatrics, University of Texas Southwestern Medical Center, Dallas, TX 75201, USA; 8 Department of Neurology and Neurotherapeutics, University of Texas Southwestern Medical Center, Dallas, TX 75201, USA; 9 School of Veterinary Medicine, Faculty of Health & Medical Sciences, University of Surrey, Guildford GU2 7AL, UK; 10 Fitzpatrick Referrals, Godalming GU7 2QQ, UK

**Keywords:** progressive myoclonic epilepsy, hypnic jerk, photoconvulsions, canine, dodecamer repeat mutation, *EPM2A*, *NHLRC1*, muscle biopsy, Chihuahua, French Bulldog, Griffon Bruxellois

## Abstract

Canine Lafora disease is a recessively inherited, rapidly progressing neurodegenerative disease caused by the accumulation of abnormally constructed insoluble glycogen Lafora bodies in the brain and other tissues due to the loss of NHL repeat containing E3 ubiquitin protein ligase 1 (*NHLRC1*). Dogs have a dodecamer repeat sequence within the *NHLRC1* gene, which is prone to unstable (dynamic) expansion and loss of function. Progressive signs of Lafora disease include hypnic jerks, reflex and spontaneous myoclonus, seizures, vision loss, ataxia and decreased cognitive function. We studied five dogs (one Chihuahua, two French Bulldogs, one Griffon Bruxellois, one mixed breed) with clinical signs associated with canine Lafora disease. Identification of polyglucosan bodies (Lafora bodies) in myocytes supported diagnosis in the French Bulldogs; muscle areas close to the myotendinous junction and the myofascial union segment had the highest yield of inclusions. Postmortem examination of one of the French Bulldogs revealed brain Lafora bodies. Genetic testing for the known canine *NHLRC1* mutation confirmed the presence of a homozygous mutation associated with canine Lafora disease. Our results show that Lafora disease extends beyond previous known breeds to the French Bulldog, Griffon Bruxellois and even mixed-breed dogs, emphasizing the likely species-wide nature of this genetic problem. It also establishes these breeds as animal models for the devastating human disease. Genetic testing should be used when designing breeding strategies to determine the frequency of the *NHLRC1* mutation in affected breeds. Lafora diseases should be suspected in any older dog presenting with myoclonus, hypnic jerks or photoconvulsions.

## 1. Introduction

Lafora disease is one of the severest forms of myoclonic epilepsies that is observed in several species but particularly in humans and dogs. It is a recessively inherited disease caused by several different mutations in genes coding for laforin glucan phosphatase (*EPM2A*) or NHL repeat containing E3 ubiquitin protein ligase 1 (*NHLRC1*, also known as *EPM2B*) [1,2,3]. Primary clinical signs include myoclonus (photomyoclonic response), tonic–clonic seizures, visual hallucinations and blindness [4,5]. Rapid progression of the disease leads to more severe and frequent seizures with increased refractoriness, ataxia, dementia and, eventually, in humans, a vegetative state. The human patient dies usually within 10 years of onset due to status epilepticus [4]. Dogs are typically euthanized when quality of life is significantly impacted by the disease [5].

Normally, the glucose storage molecule glycogen is soluble, but in the absence of *EPM2A* or *NHLRC1*, it is malstructured and becomes insoluble. This leads to the accumulation of the abnormal glycogen into Lafora bodies in neurons and astrocytes and progressive neurodegeneration [3,6]. Impairment of astrocytic function and neuroinflammation drives this neurodegeneration [7], blocking brain glycogen synthesis in mouse Lafora disease models preventing disease progression [8,9,10]. Lafora bodies accumulate in other tissues such as muscle and liver, but these organs remain unimpaired, at least in the lifespan of the canine patient [4,5].

Lafora disease in dogs is caused by a repeat expansion mutation in the NHL repeat containing the E3 ubiquitin protein ligase 1 (*NHLRC1*) gene [11,12]. Affected individuals carry 19 to 26 copies of the repeat sequence rather than the expected 2–3 copies [13]. Dogs, relative to other species, are predisposed to Lafora disease. Their genome uniquely contains a dodecamer repeat sequence within the *NHLRC1* gene, which is prone to unstable (dynamic) expansion. This canid dodecamer repeat is ancient. It is not present in felines, which split from a common Felidae and Canoidae ancestor 60 million years ago [11]. Fifty million years ago, Canidae (wolves, dogs, foxes, coyotes and jackals, etc.) split from the common ancestor for Arctoidea (bears, raccoons, otters, skunks, etc.) and Pinnipeds (seals, sea lions and walruses) [13]. Arctoidea have one copy of the dodecamer repeat, and all canids have 1–3 polymorphic copies, with the dog, wolf and coyote having 2–3 copies [11]. Separation of Canidae into 35 extant species occurred relatively recently at about 10 million years ago [13]. This suggests that there were two events associated with the canid dodecamer repeat, one after Felidae split from the common ancestor 60 million years ago and one after Canidae split from other members of the superfamily Canoidae 50 million years ago. The canid dodecamer repeat is unstable, meaning that it is prone to expansion in successive generations. This expansion mutation prevents transcription and, when biallelically present, leads to loss of function of the gene and Lafora disease [11,12]. As such, the tendency for Lafora disease is a species-wide problem that will likely appear spontaneously in many breeds and even crossbreeds. So far, Lafora disease due to *NHLRC1* dodecamer repeat expansion mutation has been reported in Miniature Wirehaired Dachshund [11], Basset Hound [11], Beagle [12,14], Chihuahua [15], Pembroke Corgi [16] and the following breeds on the basis of histology: Miniature Poodle [17], Standard Poodle [18] and Pointer [19]. However, because of the unstable dodecamer repeat, Lafora disease may occur in any purebred or mixed-breed dog. Due to the severity of the disease, the dogs might be euthanized before confirmed diagnosis, and Lafora disease might not be recognized. In addition, veterinary surgeons may not submit testing because they assume that the disease is unlikely because the affected dog is not a predisposed breed.

In this study, we describe Lafora disease and management in three dog breeds, the Chihuahua, French Bulldog and Griffon Bruxellois, but also in a crossbreed dog with Chihuahua, Yorkshire terrier and Chinese Crested dog genetic ancestry. Diagnosis was based on clinical and histological data. Genetic testing confirmed the presence of the *NHLRC1* repeat expansion mutation. These three breeds represent model breeds for Lafora disease. This and the identification in a mixed-breed dog reinforce the conclusion that the disease is not likely to be confined to a small number of purebred dogs but is a species-wide tendency.

## 2. Materials and Methods

### 2.1. Patient Cohort

Case 1: A 7-year-old male neutered Chihuahua was presented to the Neurology Service of the University of Liverpool for further investigation of acute onset epileptic seizure-like activity with onset 2 months previously. The owner reported an isolated generalized tonic–clonic epileptic episode. Additionally, the dog had daily, frequent, sudden head and body movements characteristic of myoclonic jerks (normal consciousness, no autonomic signs), which could be triggered by bright light, loud noises and movement toward the head. 

After clinical examination and routine blood work, the dog underwent brain magnetic resonance imaging (MRI). The images were acquired with a 1.5 T Magnetom Harmony (Siemens, UK), and the sequences were obtained according to Rusbridge et al. [20]. Routine cerebrospinal fluid (CSF) analysis was performed. The dog was prescribed levetiracetam (22 mg/kg *per os* q8 h Keppra suspension, GlaxoSmithKline) and an antioxidant and essential fatty acid rich commercially available diet (Purina ProPlan Bright Minds™). Phenobarbital (2.5 mg/kg *per os* q12 h; Epiphen, Vetoquinol) treatment was added.

Case 2: A 7-year-old female French Bulldog was presented to the Small Animal Clinic Hofheim with reflex myoclonus initially triggered by rapid light/shadow changes, followed by increasingly severe and frequent myoclonic phases. Diagnostic workup included general and neurological examination, ophthalmic examination, complete blood work, brain MRI and CSF analysis. The MRI images before and after intravenous gadolinium contrast medium were acquired with Toshiba’s Vantage Elan 1.5 T system. The dog was prescribed imepitoin (11.5 mg/kg *per os* q12 h; Pexion, Boehringer Ingelheim) and phenobarbital (2.3 mg/kg *per os* q12 h; Epiphen, Vetoquinol).

Case 3: A 9.5-year-old female French Bulldog was presented to the Small Animal Clinic Hofheim with first clinical signs described as a startle response observed at rest and in dim light. Diagnostic workup was performed as with Case 2, and in addition, a muscle biopsy was performed. Imepitoin (11.3 mg/kg *per os* q12 h Pexion, Boehringer Ingelheim) was prescribed. Due to lack of response levetiracetam was added (18.8 mg/kg *per os* q8 h Keppra suspension, GlaxoSmithKline).

Case 4: A 12-year-old male neutered Griffon Bruxellois was presented to Lumbry Park Veterinary Specialists, UK, with a 13-month history of progressive clinical signs of myoclonus, photosensitivity, vision loss and behavioral changes. Diagnostic work up included general and neurological exam, ophthalmic examination, blood work and genetic testing for a mutation in the NHLCR1 gene. The client declined advanced imaging with MRI, CSF analysis or tissue biopsy.

Case 5: A 7-year-old male neutered mixed-breed dog presented to Highcroft Veterinary Referrals, UK, with a 3-month history of hypnic jerks (Video S1). The hypnic jerks were of such intensity that they disrupted sleep. Other spontaneous or reflex myoclonus had not been observed. Diagnostic work up included general and neurological examination, blood work, brain MRI (Philips Achieva 1.5T system), CSF analysis and genetic testing for the mutation in the NHLCR1 gene. Levetiracetam (22 mg/kg *per os* q8 h) was prescribed with an antioxidant-rich, low-carbohydrate diet. Breed ancestry was investigated using the commercially available Wisdom Panel ^TM^ test (Vancouver, WA, USA).

### 2.2. Patient Consent and Ethical Committees

This study followed international, national and institutional guidelines for humane animal treatment and complied with relevant legislation. This study involved client-owned animals and demonstrated a high standard (best practice) of veterinary care. Informed client consent was obtained for inclusion of study subjects. Canine Lafora genetic testing was performed under the Ethical Committee Approval VREC589.

### 2.3. DNA Testing

Canine Lafora genetic testing was performed on whole-blood samples collected from the affected Chihuahua (Case 1) and French Bulldog (Case 3) and breed-matched, clinically healthy, random control dogs (6 Chihuahuas and 7 French Bulldogs).

Genomic DNA was extracted from whole blood in EDTA using phenol-chloroform extraction. Concentration was measured using NanoDrop (Thermo Fisher, Waltham, MA, USA) and stored at − 20 °C. Genomic DNA (10 μg) was digested with DraIII (New England Biolabs #R3510, Ipswich, MA, USA) and EcoRI (New England Biolabs #R0101 L) overnight. Genomic DNA was separated on 1% agarose gel and nicked with 0.3 M HCl. The DNA was denatured with 0.5 M NaOH + 1.5 M NaCl and neutralized with 0.2 M Tris (pH 7.4), before transferring to Hybond-N membrane (Amersham Hybond N+). The membrane was hybridized with a P32-labeled DNA fragment specific to canine *NHLRC1* (GenBank Number.: AY560905.1, CanFam3.1, chr35:16,920,972–16,921,534). The membrane was washed several times with 1 M sodium phosphate and 20% SDS, and the membrane was exposed onto X-ray film. Dogs with normal *NHLRC1* alleles have one 986 bp band, affected dogs with the dodecamer expansion have a band close to 1500 bp depending on the number of repeats [11] and heterozygous carriers have one normal and one mutated allele. As the dodecamer repeat expansion mutation is variable, Southern blot is considered the most accurate method to distinguish different genotypes. Genetic testing for Cases 4 and 5 was performed using whole blood in EDTA using a validated PCR technique by the commercial laboratory Laboklin UK (125 Northenden Road, Manchester, UK).

### 2.4. Histopathology

Representative brain samples were collected from Case 2, fixed with 10% neutral buffered formalin and processed as described elsewhere [21]. Selected areas were embedded in paraffin and stained with hematoxylin and eosin (H&E), with periodic acid-Schiff (PAS) staining with and without diastase pretreatment. Muscle tissue biopsies were collected from Cases 2 and 3. They were processed for cryohistology after mounting with an optimal cutting temperature compound (TissueTek^®^, Sakura Finetek USA, Inc. Torrance, CA, USA.) and snap freezing in isopentane cooled in liquid nitrogen. Cryosections were accompanied by paraffin sections of formalin-fixed muscle fragments, and both types of sections underwent the staining mentioned above.

## 3. Results

### 3.1. Clinical Findings

#### 3.1.1. Case 1: Chihuahua

Physical and neurological examinations were considered normal except for visible myoclonic jerks. Based on the age, history and characteristics of the dog’s episodes, forebrain localization and Lafora disease were suspected.

Hematology, biochemistry, including glucose levels (5.1 mmol/L (reference interval (RI): 3.6–7))) and fasting bile acids (2.4 mmol/L (RI: 0.1–5)), were within normal limits. Magnetic resonance imaging of the brain revealed the presence of Chiari-like malformation and a partially empty sella/pituitary cyst. Both findings were considered conformational in origin and not clinically relevant. CSF (cerebellomedullary sample) revealed a normal cell count (0 cells/µL (RI: <5/µL)) and protein concentration (0.31 g/L (RI: 0.35)).

The dog did not tolerate the use of Doggles^TM^ (Diamond Springs, CA, USA) for photosensitivity. Despite slight improvement for several weeks following the prescription of levetiracetam and an antioxidant-rich diet, the dog continued to experience frequent myoclonic jerks. Phenobarbital treatment was then added to the therapy and subsequently increased to 5 mg/kg *per os* q12 h. The dog remained stable but experienced frequent myoclonic jerks and occasional (less than one per month) generalized tonic–clonic epileptic seizure activity. The medication was well tolerated aside from slight sedation at subtherapeutic phenobarbitone serum concentration at 11.4 mg/L (RI: 20–35, IDEXX Laboratories, Wetherby, UK). The dog was reported to be doing well 1.5 years after the onset of clinical signs.

#### 3.1.2. Case 2: French Bulldog

**Figure 1:**
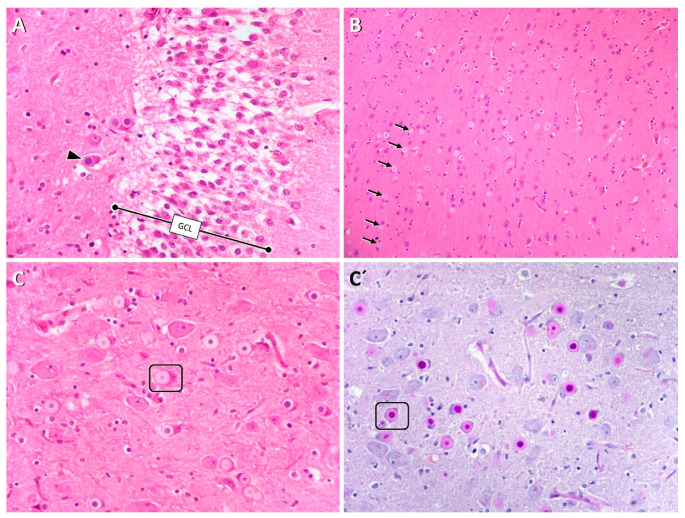
Somatodendritic Lafora bodies were particularly prominent in the hilus of the dentate gyrus (**A**, arrow), bordered by the inner surface of the granule cell layer (**A**, GCL), in pyramidal cells of the neocortex (**B**, arrows) and in the lateral geniculate nucleus (**C**,**C′** frame). The core of Lafora bodies stain basophilic on H&E (**A**–**C**) and purple on PAS (**C**).

All clinical and neurological examinations were within normal limits except for the tendency for myoclonus. There was no clinical improvement following the prescription of antiepileptic drugs imepitoin and phenobarbital. The dog was euthanized about one year after the onset due to the high frequency of myoclonic jerks and impact on quality of life. Tissues were collected for postmortem analyses. Histology of the brain revealed intraneuronal accumulation of somatodendritic polyglucosan bodies (Lafora bodies) throughout virtually all gray-matter areas. The inclusions were characterized by a pale homogenously amphophilic (H&E) and purple (PAS) periphery surrounding a round to spheric basophilic (H&E) and diastase-resistant PAS-positive core, as depicted in Figure 1. Similar but elongated inclusions were identified in myocytes from various skeletal muscles. Notably, muscle areas close to myotendinous junction and the myofascial union segment presented with the highest yield of inclusions.

#### 3.1.3. Case 3: French Bulldog

All clinical and neurological examinations were within normal limits apart from the tendency for myoclonus. Imepitoin therapy reduced the myoclonic episodes, which remained at a stable frequency for about 7 months. Muscle biopsies confirmed the presence of sarcoplasmic polyglucosan bodies within the striated muscle fibers. Interestingly, a five-day course of nonsteroidal anti-inflammatory drugs given after biopsy led to reduction of the frequency of myoclonus, which increased again when therapy was withdrawn. Following this, levetiracetam was prescribed in addition to imepitoin, resulting in a significant reduction of myoclonus frequency, and former triggers, for example watching television, no longer induced myoclonus. Two and a half years after initiation of levetiracetam therapy, the dog is in a stable neurological condition at a dosage of 22 mg/kg *per os* q8 h in combination with imepitoin at 15 mg/kg *per os* q12 h.

#### 3.1.4. Case 4: Griffon Bruxellois

General physical examination documented a grade 4/6 systolic heart murmur that had previously been investigated by a European diploma holding cardiologist and reported as being secondary to mitral valve degenerative disease. Neurological examination documented myoclonic episodes in response to clapping and the menace response test. Blood work showed an unremarkable complete blood count, and biochemistry (including NH_3_) showed borderline elevation in ionized calcium of 1.42 mmol/L (RI: 1.12–1.40), urea of 8.0 mmol/L (RI: 1.7–7.4) and ALP of 160 U/L (RI: 12–83). None of these changes were considered likely to account for the case’s clinical signs. *Toxoplasma* and *Neospora* serology revealed a positive *Toxoplasma* IgG antibody titer at a dilution of 1:50 but negative IgM at a dilution of 1:20. This was suggestive of previous exposure and not active infection. Follow-up sampling of the *Toxoplasma* serology levels was requested but declined by the owner. Levetiracetam and an antioxidant-rich commercially available diet and Doggles^TM^ were prescribed. Following the prescription of levetiracetam (17.8 mg/kg *per os* q8 h; Glenmark Pharmaceuticals Europe Ltd. Watford, UK), the myoclonic episodes ceased for 3 months but then restarted at a low frequency, and the case continued to show progressive behavioral changes thought to be due to cognitive decline. The breeder of Case 4 reported that one female sibling also had signs of Lafora disease that the mother and two other siblings were unaffected. The mother had died aged 15 years.

#### 3.1.5. Case 5: Mixed-Breed Dog

**Figure 2:**
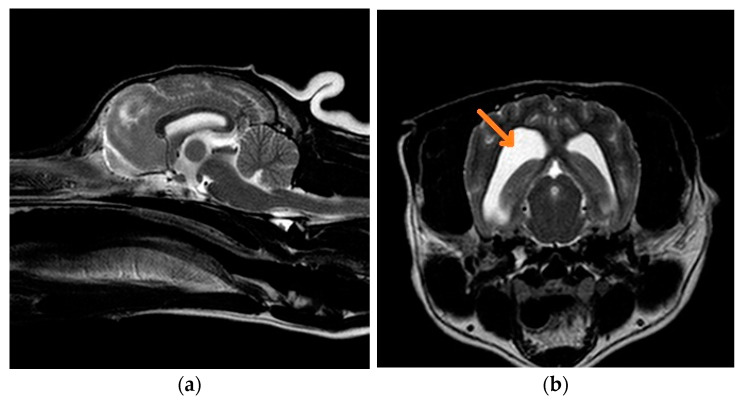
(**a**) Midsagittal T2W MRI from a 7-year-old mixed-breed dog with Lafora disease; (**b**) transverse T2W MRI at the level of the temporal lobe from a 7-year-old mixed-breed dog with Lafora disease. There is dilatation of both lateral ventricles (orange arrow), which can be an early indicator of cortical atrophy associated with the disease. However, the size of the intrathalamic adhesion and subarachnoid space is normal.

Physical and neurological examinations were normal. Blood work documented an unremarkable complete blood count. Biochemistry showed a moderate increase in ammonia of 71 µmol/L (RI: 11–54), which was considered unlikely to explain the clinical signs. Magnetic resonance imaging of the brain (1.5T Achieva 1.5 DS MR system, Philips, Farnborough, UK) revealed asymmetrical lateral ventriculomegaly (Figure 2); the significance of this was equivocal and may be related to a brachycephalic skull type. Measurement of the intrathalamic adhesion and subarachnoid space was normal. CSF analysis (cerebellomedullary sample) revealed a normal cell count (1 cell/µL (RI: <5/µL)) and protein concentration (0.2 g/L (RI: <0.35)). The dog markedly improved following the prescription with levetiracetam and an antioxidant-rich, low-carbohydrate diet. Breed ancestry according to the commercial Wisdom panel ™ was 37.5% Yorkshire terrier, 25% Chihuahua, 25% Chinese Crested dog and 13% terrier breed group.

### 3.2. DNA Testing

**Figure 3:**
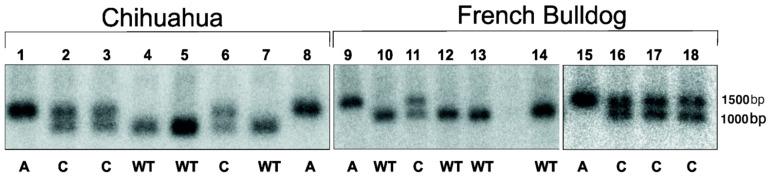
Genotyping using Southern blot of the Chihuahua (1) and French Bulldog (9) suspected to be affected with Lafora disease. Control dogs, Chihuahuas (2–7) and French Bulldogs (10–14, 16–17) with no clinical signs were included. Previously genotyped MWHDs with known clinical status, Lafora disease affected (8, 15) and unaffected carrier (18) were used as genotyping controls. The affected dogs (**A**) are homozygous for the mutation, carriers (**C**) have one normal and one mutated allele and clear dogs (**WT**) have normal three copies of the repeat.

Lafora disease was suspected based on the clinical and neurological signs. Postmortem examination using PAS and H&E staining indicated the presence of Lafora bodies in Case 2. Genetic DNA testing was performed for the known *NHLRC1* mutation for Cases 1, 3, 4 and 5; all dogs were homozygous for the disease mutation. In addition, blood samples from breed-matched, clinically healthy, random control dogs were collected. Moreover, samples from Miniature Wirehaired Dachshunds with known genotypes and clinical status were used as genotyping controls (homozygous affected and heterozygous unaffected). The Chihuahua and the French Bulldog with clinical signs of Lafora disease were homozygous for the dodecamer repeat expansion, which confirmed they were affected with canine Lafora disease (Figure 3). The breed-matched clinically healthy, random control dogs were either carriers, heterozygous or clear, homozygous for the normal allele. Cases 4 and 5 had genetic testing using whole blood in EDTA using a validated PCR technique by the commercial laboratory Laboklin UK (125 Northenden Road, Manchester, UK) and were homozygous for the *NHLRC1* mutation. The reason for the difference in genetic testing was the unavailability of the southern blot technique during the relocation of the author BAM’s laboratory.

## 4. Discussion

Lafora disease is a glycogen storage disease that manifests as a progressive myoclonic epilepsy that aggravates with age. The loss of function of the *NHLRC1* gene leads to abnormal glycogen synthesis. Normally, the glucose storage molecule, glycogen, is soluble. During glycogen synthesis, while glycogen synthase (GYS1) extends glucan chains through α1–4 linkages, every six units added are removed by glycogen branching enzyme (GBE1) and reattached upstream through an α1–6 bond, growing the molecule radially into a sphere. Precisely how the sphericity of glycogen is ensured remains unknown, though it is clear that this is managed through a tight regulation by laforin of a small amount of covalently bound phosphate on glycogen [22,23,24,25]. The malstructured insoluble glycogen (polyglucosan) gradually precipitates over months and years and accumulates into Lafora bodies, which cannot be digested by the normal glycogen-digesting enzymes.

The clinical signs of canine Lafora disease are similar to the human disease and include generalized tonic–clonic epileptic seizures, myoclonus, photosensitivity and hypnic jerks [5,11]. Classically, myoclonus is triggered by movement close to the head, noise or bright light and can also be spontaneous. The muscle contractions range from contraction of the periorbital and other facial muscles with a backward movement of the head to a violent jerking of the extremities with a backward “jump”.

The dogs may suffer from “panic attacks”, atypical and novel signs of fear and escape behavior, which may be an indication of visual hallucinations. As the disease progresses, the myoclonus and the epileptic seizures become more severe, and the dog shows signs of ataxia and increasing signs of cognitive decline and visual loss. The dogs are usually euthanized within a few years of onset due to the severity of clinical signs and the impact on quality of life [5,11,14,26].

The dogs presented to the veterinary neurologists showed signs of a progressive neurodegenerative disease and classical clinical signs of Lafora disease, including myoclonus, tonic–clonic seizures, photosensitivity, vision loss and hypnic jerks. The clinical signs and age of onset corresponded to the previously reported clinical signs in other breeds like the Miniature Wirehaired Dachshund [5]. The presence of typical PAS-positive aggregates in the brain and muscle supported Lafora disease for Cases 2 and 3. Muscle histology should be considered as an ancillary intravital test for polyglucosan body diseases and in particular for breeds for which genetic tests are not available or have not been validated sufficiently. Muscle tissue adjacent to myotendinous insertion and the myofascial union are most likely to feature enough inclusions that are only sparsely scattered in the muscle belly area.

MRI findings and results of the CSF tap were unremarkable in both French Bulldog cases; in the Yorkshire terrier/Chihuahua/Chinese Crested mixed-breed dog MRI may have indicated early cortical atrophy.

The canine *NHLRC1* gene, unlike human or feline genes, contains 2–3 copies of a dodecamer repeat, which is prone to expansion [11]. The genetic testing of this repeat in the Chihuahua, French Bulldog, Griffon Bruxellois and mixed-breed dog confirmed the presence of dodecamer repeat expansion mutation in *NHLRC1* previously reported in several other breeds affected with Lafora disease [11,12]. The clinically healthy dogs were either carriers or had two normal alleles of the gene. In Chihuahuas, 50% (3/6) and 43% (3/7) of French Bulldog controls carried the mutation. This might indicate that the carrier frequency in these breeds might be high, but this warrants further studies in larger sample cohorts.

Currently, there is no curative treatment for Lafora disease, and disease management is limited to controlling the myoclonus and seizures [27]. Levetiracetam can be useful to reduce the myoclonus in the early stages of the disease and was used to reduce myoclonus in Cases 1, 2, 4 and 5. Levetiracetam is a new antiepilepsy drug with an antimyoclonic effect, especially when the myoclonus is cortical in origin [28,29]. The mechanism by which the accumulation of Lafora bodies results in a myoclonic epilepsy is still debated, but there is increasing evidence that the accumulation of glycogen inclusion in astrocytes is paramount [30]. Astrocytic function is affected along with a progressive neuroinflammatory response and oxidative stress [30,31,32]. It is hypothesized that inflammation, astrocyte reactivity and activation of microglia play a major role in triggering seizures [31]. In a mouse model of the diseases, this progressive increase in inflammatory response correlates with age, providing a possible explanation for the worsening of the pathophysiological clinical signs over time [31]. Improved understanding of the pathophysiology of Lafora’s disease has sparked the possibility of new treatment strategies, as reviewed by Sanz and Serratosa [33]. Interestingly for both humans and dogs, the first clinical signs of Lafora disease occur at a similar chronological age (in dogs at seven years of age and in humans during late childhood/early adolescence). The disease then progresses at a similar rate over the remaining lifetime of the dog and over 10 years in a human. This is exceptional for a canine model of a human disease. In all other neurodegenerative diseases, the equivalent disease in canines occurs at the same epigenetic age [34], i.e., a disease affecting an adolescent human will occur in a puppy aged 8–10 months.

The evidence of increased oxidative stress due to a glycogen storage disease means that many veterinary surgeons recommend a proprietary antioxidant-rich, low-carbohydrate diet in the management of Lafora disease [35]. However, there is poor evidence that this alters the disease course [5]. A lifelong ketogenic diet reduces Lafora bodies in a mouse model of Lafora disease; however, human studies were less promising but did suggest that the disease may progress more slowly if a ketogenic diet was started early in the disease process [36,37]. A ketogenic diet aims to maintain blood glucose concentration in the low normal range and the brain uses more keto acids for energy production. A ketogenic diet is also antiepileptic due to γ-glutamyl transpeptidase and γ-glutamylation inhibition [37]. There is one commercial medium-chain triglyceride ketogenic diet (Purina Neurocare™) that has achieved clinically meaningful levels of ketosis in dogs and helped prevent seizures and improve cognition in dogs with idiopathic epilepsy [38,39,40]. This diet has not been trialed for Lafora disease, but based on the available evidence, this or a similar diet is probably the most appropriate for dogs with Lafora disease. However, not all dogs or owners accept a change in diet, especially if the perceived benefit is not obvious or unproven.

If the dog develops tonic–clonic seizures, then the addition of the antiepilepsy effective against generalized seizures is indicated. Phenobarbital and imepitoin had no or little effect for Cases 1–3. Anecdotally one of the authors (CR) finds zonisamide more useful for the management of generalized seizures in Lafora disease. Some owners of Lafora disease-affected dogs choose to medicate with cannabidiol (CBD) oil; however, studies in rodent models found that CBD-enriched extract did not reduce the severity of the epileptic seizures, although it may reduce the cognitive impairment [41].

## 5. Conclusions

The tendency for Lafora disease is a species-wide problem that will likely appear spontaneously in many breeds and mixed-breed dogs. We describe here three dog breeds and a mixed-breed dog affected with Lafora disease. Diagnosis was confirmed with DNA testing and histopathology. This is the first report of progressive myoclonic epilepsy in French Bulldogs and the Griffon Bruxellois and establishes these breeds as an additional canine model for Lafora disease. As Lafora disease is a severe neurodegenerative disease, genetic testing should be considered when designing breeding strategies in these breeds. This is particularly important for the Griffon Bruxellois breed, which has a small genetic pool. As Lafora disease is late onset, affected dogs may have been bred well before developing clinical signs. This and the asymptomatic carrier state mean that the disease has the potential to become widespread within closed populations. DNA testing could also be used to determine the frequency of the mutation and possibly eradicate Lafora disease from these breeds. Muscle biopsy is a useful ancillary test especially for breeds where genetic tests are not available or have not been validated sufficiently. The biopsy should be from muscle tissue adjacent to myotendinous insertion and the myofascial union as Lafora bodies are only sparsely scattered in the muscle belly area.

## Supplementary Materials

The following are available online at https://www.mdpi.com/article/10.3390/life11070689/s1, Video S1: Hypnic jerks in a mixed-breed dog with Lafora disease.
